# Culture-negative bivalvular endocarditis with myocardial destruction in a patient with systemic lupus erythematosus: a case report

**DOI:** 10.1186/1749-8090-6-109

**Published:** 2011-09-14

**Authors:** Brett R Laurence, Byungse Suh

**Affiliations:** 1 Section of Infectious Diseases, Temple University School of Medicine, Philadelphia, Pennsylvania, USA

## Abstract

Culture-negative endocarditis has long been associated with systemic lupus erythematosus, but is usually asymptomatic or involves a single valve. We present a patient with destructive culture-negative endocarditis that remains without a microbial etiology despite an exhaustive workup using advanced diagnostic techniques in a patient with systemic lupus erythematosus.

## Background

Culture-negative endocarditis (CNE) is known by many names including marantic endocarditis (ME), non-bacterial thrombotic endocarditis, verrucous endocarditis, and Libman-Sacks vegetations in collagen vascular diseases, specifically, systemic lupus erythematosus (SLE). First described by Zeigler [1] in 1888 and derived from the Greek marantikos, meaning "wasting away", ME typically involves a single valve with rare involvement of two or more valves [2]. Structural valve disease is common in the SLE population and the valve abnormality usually consists of leaflet thickening with small vegetations often discovered at autopsy [2,3]. The pathophysiology of vegetation formation is not entirely understood, but involves platelet deposition on a damaged endothelial surface, possibly from up-regulated cytokines and immune complex damage, with an absence of inflammatory cells [3,4]. Though typically asymptomatic, there is an excess incidence of stroke, embolism, and heart failure. Valvular lesions appear to be unrelated to duration or activity of illness and may occur at any time [2]. There are few cases of multi-valvular involvement with ME and even fewer cases that involve direct myocardial damage. We present the case of a woman with SLE admitted for an elective mitral valve repair who was found to have mitral and aortic valve culture-negative vegetations with atrial destruction. A thorough workup for a possible microbial etiology utilizing current advanced techniques was negative.

## Case Presentation

A 42 year old woman with SLE for the past 12 years and end stage renal disease requiring peritoneal dialysis was admitted to the hospital for congestive heart failure. Her SLE was controlled on hydroxychloroquine and prednisone 10 mg daily for the past 5 years. Prior to admission, she had a long-standing IV/VI systolic murmur, and a transthoracic echocardiogram revealed severe mitral regurgitation with a left ventricular ejection fraction of 35%. A subsequent transesophageal echocardiogram showed mild mitral valve thickening without vegetations and normal aortic, tricuspid, and pulmonic valves. Three months later as she was approaching the date for her elective mitral valve repair, she was admitted with 3 days of progressive dyspnea and severe, left sided chest pain radiating to her back. Physical examination showed a thin woman without hypotension or hypoxia. Her heart rate was 95 bpm and she had the same systolic murmur. She also had bilateral pulmonary crackles. She had a diffuse hyperpigmented mottled rash over her extremities, back, and trunk without stigmata of endocarditis.

She had the following lab results with normal ranges shown in brackets when values were abnormal: a hemoglobin of 8.2 g/dL [11.5 - 16.0 g/dL], white blood cell count 8.4 K/mm^3^, platelets 240 K/mm^3^, creatinine phosphokinase 95 U/L, myoglobin 3.4 ng/mL, cardiac troponin I 0.26 ng/mL. Electrolytes were normal and her blood urea nitrogen was at baseline of 53 mg/dL [10-20 mg/dL]. The ferritin level was 2055 ng/mL [10 - 29 ng/mL]. The EKG was unchanged from before. She had a loculated effusion within the minor fissure without pneumonia.

A new transesophageal echocardiogram revealed severe aortic insufficiency with destruction of the right coronary and non-coronary cusps, severe mitral insufficiency with destruction of the anterior leaflet, a fistula between the aorta and left atrium, and a left ventricular ejection fraction of 35%. In the operating room, it was clear that the right ventricle and right atrium were enmeshed in a dense inflammatory "phlegmon" extending to the aortic root. The right coronary and non-coronary cusps of the aortic valve were replaced by vegetations. The anterior mitral leaflet showed a large vegetation containing pus down to the head of the papillary muscle. The patient required an aortic valve replacement, mitral valve replacement, and reconstruction of the superior vena cava, dome of the left atrium, right atrium, and intra-atrial septum.

**Figure 1:**
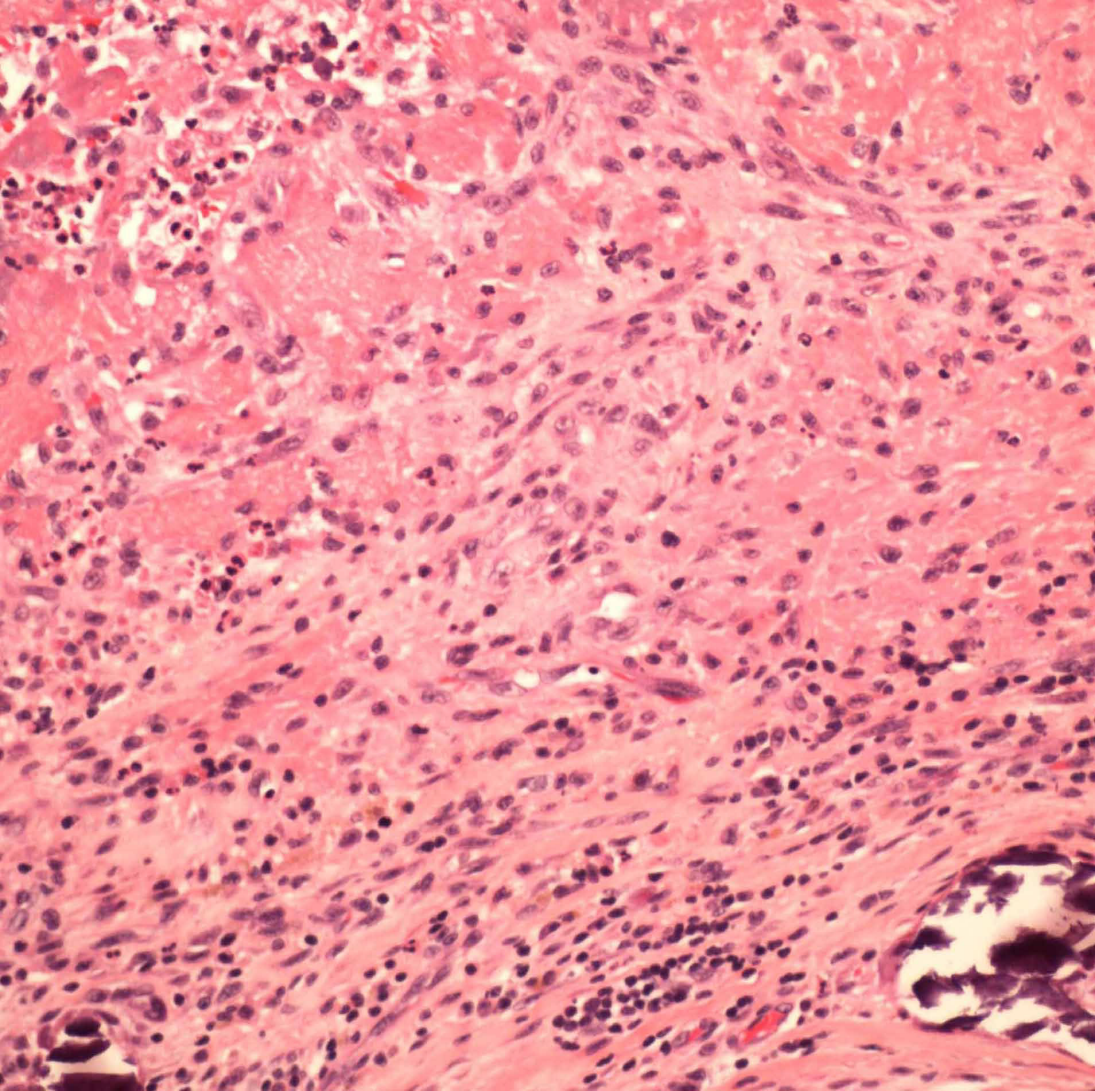
**Hematoxylin and eosin (H&E) stain of aortic valve specimen demonstrating fibrin, neutrophils, and calcification**.

Valve tissue was sent for pathology and microbiologic analysis. The patient was started on vancomycin and ciprofloxacin. The post-operative course was uneventful: she remained afebrile and was easily extubated on day 3. Gram stain of the valvular tissue demonstrated no white blood cells and no organisms; cultures for bacteria (retained for 14 days), fungi and mycobacteria were all negative. Histopathologic examination of the valves revealed extensive fibrin, neutrophils, and calcification suggestive of infective endocarditis (Figure 1). Fungal and acid fast stains were negative. The patient was discharged on doxycycline for presumptive culture-negative endocarditis. Serologies for *Coxiella burnetii *and *Brucella melitensis *were negative. At follow-up four months later she had no bacteremia, and a repeat transthoracic echocardiogram revealed normal appearing aortic and mitral bioprostheses. Tests for *Legionella *were not performed during the initial evaluation though a urine *Legionella *antigen was negative at follow-up five months later.

The valve tissue was sent for broad-range polymerase chain reaction (PCR) amplification to Unite des Rickettsies, Faculte de Medecine, Universite de la Mediterranee in Marseille, France for the following agents: *Bartonella *species, *T. whipplei*, *C. burnetii*, *Mycoplasma *species, fungi, *Streptococcus/Enterococcus *species, and *Staphylococcus *species which were all negative. In addition, immuno-histochemistry for *Bartonella *species and *C. burnetii *were also negative.

## Discussion

This case raises the possibility of an alternate understanding of marantic or Libman-Sacks endocarditis. Our patient had no evidence of active infection prior to admission, and a subsequent workup including extended culture duration, serologies, histopathological examination, and PCR did not reveal a microbial etiology despite the degree of purulent destruction.

Infective endocarditis (IE) associated with three or more negative blood cultures (culture-negative endocarditis) constitutes 5% of all endocarditis cases [5,6]. The reasons for culture negativity are related to technical limitations of culture (e.g. not using specialized media, antibiotic administration prior to obtaining blood cultures) or to the specific organism (e.g. fungi, fastidious bacteria), though HACEK group bacteria - formerly considered a common cause of culture-negative endocarditis - are usually isolated within 5 days with current blood culture systems [5-7].

Difficult-to-cultivate microorganisms including *T. whipplei*, *Bartonella *spp., *C. burnetti*, *Legionella *spp., and *Mycobacterium *spp., have been identified with serologic testing and PCR amplification [8]. In a study of almost 2,000 patients with clinically suspected endocarditis, 21% had definite endocarditis and 13% had possible endocarditis by Duke criteria while the remainder were rejected [6]. In the definite endocarditis group, an etiology was established in over 90% by blood and valve tissue culture, serological testing, and PCR of valve tissue [6]. Culture negative endocarditis is less well documented by the Duke criteria than culture positive endocarditis [9]. The reason for this difference lies, in part, with the use of positive blood cultures as a key component of the Duke criteria. Histopathology of valve tissue can also be useful in differentiating myxoma, rheumatic endocarditis, and marantic endocarditis from IE.

Broad-range PCR analysis targets commonly shared bacterial 16S *rRNA *genes (18S *rRNA *for fungi) through the use of primers. Bosshard et al compared broad-range PCR to standard microbiological techniques (Gram staining and culture) on endocardial specimens from 49 patients with good overall agreement, but 18% of patients with negative blood cultures were positive with broad range PCR [8]. PCR provided a higher diagnostic yield and was much less affected by prior administration of antibiotics [8]. Houpikan and Raoult performed etiologic testing on sera, blood, and valve tissue from 348 patients with infective endocarditis in France using culture in shell vial, indirect immunofluorescent antibodies, histopathology, and PCR amplication [5]. Five patients had rare bacteria (including *T. whipplei, M. hominis, Abiotrophia elegans*, and *Legionella pneumophila*). *C. burnetii *is more common in France than in the United States and represented a majority (48%) of the IE diagnoses in this cohort [5].

A more recent study by Fournier and Raoult consisted of specimens obtained from over 750 blood culture negative endocarditis patients [10]. Specimens underwent testing incorporating serological, molecular, and histopathological analysis including culture and PCR of cardiac valve tissue. While the majority received antibiotics prior to cultures; serologic analysis using immunofluorescence assay provided a diagnosis in almost half the patients (mostly *C. burnetii *followed by *Bartonella *species) and PCR of valvular biopsies diagnosed over 60% including 109 patients for whom serological results were negative [10]. PCR had a higher yield from valve tissue than from blood specimens with high sensitivity for *Bartonella *species, *C. burnetti*, and *T. whipplei *[10]. Among 115 patients without a documented infection, 2.5% had non-infective endocarditis including marantic endocarditis, collagen vascular diseases, angiosarcoma, and atrial myxoma [10]. Noted limitations of PCR include contamination of tissue with amplification of background sequences leading to false positive results. In our case, a falsely negative PCR result might have been accounted for by unavailable PCR primers for a specific organism or by processing errors resulting in sample degradation.

There are a few limitations of our patient's initial workup that may have masked a causative agent. *Legionella *testing was not performed until months after surgery and she received treatment with ciprofloxacin and doxycycline prior to testing. In addition, culture and PCR testing for nutritionally variant streptococci (NVS) such as *Abiotrophia *species was not performed and the patient received a brief course of vancomycin during her hospital course.

While the etiology of culture negative endocarditis differs regionally, the incidence of fastidious zoonotic agents is higher in developing countries. Our region has a relatively low incidence of zoonotic agents and standard culture techniques are generally adequate to detect the etiology of endocarditis. While marantic endocarditis lacks inflammatory cells and normally involves a single valve, purulence was noted on gross inspection and histopathology.

We present a patient with extremely destructive culture negative endocarditis that remains without a definitive etiologic agent despite sophisticated and advanced technological efforts. Despite limited anti-microbial therapy, the patient has shown no evidence of relapse further strengthening the case for a non-microbial cause. To date there are no reports of such devastation attributed to marantic or Libman-Sacks endocarditis, but we raise the possibility that this could be the case.

## Consent

Written informed consent was obtained from the patient for publication of this case report and accompanying images. A copy of the written consent is available for review by the Editor-in-Chief of this journal.

## Competing interests

The authors declare that they have no competing interests.

## Authors' contributions

BRL participated in the care of the patient and wrote the initial manuscript. BS participated in the care of the patient and edited the manuscript. All authors participated in approving the final manuscript.
